# A double-blind, randomised, placebo-controlled parallel study to investigate the effect of sex and dietary nitrate on COVID-19 vaccine-induced vascular dysfunction in healthy men and women: protocol of the DiNOVasc-COVID-19 study

**DOI:** 10.1186/s13063-023-07616-2

**Published:** 2023-09-16

**Authors:** Asad Shabbir, Ismita Chhetri, Rayomand S. Khambata, Tipparat Parakaw, Clement Lau, Muhammad A. B. N. Aubdool, Gianmichele Massimo, Nicki Dyson, Vikas Kapil, Thomas Godec, Vanessa Apea, Jan Flint, Chloe Orkin, Krishnaraj S. Rathod, Amrita Ahluwalia

**Affiliations:** 1 Barts and The London Faculty of Medicine and Dentistry, Queen Mary University of London, London, EC1M 6BQ UK; 2 Department of Cardiology, Barts Heart Centre, St. Bartholomew’s Hospital, Barts Health NHS Trust, London, UK; 3 Barts Health NHS Trust, The Royal London Hospital, London, UK

**Keywords:** COVID-19, Endothelium, Inflammation, Nitrate, Sex, Vascular

## Abstract

**Background:**

Cardiovascular events, driven by endothelial dysfunction, are a recognised complication of COVID-19. SARS-CoV-2 infections remain a persistent concern globally, and an understanding of the mechanisms causing endothelial dysfunction, particularly the role of inflammation, nitric oxide, and whether sex differences exist in this response, is lacking. We have previously demonstrated important sex differences in the inflammatory response and its impact on endothelial function and separately that the ingestion of inorganic nitrate can protect the endothelium against this dysfunction. In this study, we will investigate whether sex or a dietary inorganic nitrate intervention modulates endothelial function and inflammatory responses after the COVID-19 vaccine.

**Methods:**

DiNOVasc-COVID-19 is a double-blind, randomised, single-centre, placebo-controlled clinical trial. A total of 98 healthy volunteers (49 males and 49 females) will be recruited. Participants will be randomised into 1 of 2 sub-studies: *part A* or *part B*. *Part A* will investigate the effects of sex on vascular and inflammatory responses to the COVID-19 vaccine. *Part B* will investigate the effects of sex and dietary inorganic nitrate on vascular and inflammatory responses to the COVID-19 vaccine. In *part B*, participants will be randomised to receive 3 days of either nitrate-containing beetroot juice (intervention) or nitrate-deplete beetroot juice (placebo). The primary outcome for both sub-studies is a comparison of the change in flow-mediated dilatation (FMD) from baseline after COVID-19 vaccination. The study has a power of > 80% to assess the primary endpoint. Secondary endpoints include change from baseline in inflammatory and leukocyte counts and in pulse wave analysis (PWA) and pulse wave velocity (PWV) following the COVID-19 vaccination.

**Discussion:**

This study aims to evaluate whether sex or dietary influences endothelial function and inflammatory responses in healthy volunteers after receiving the COVID-19 vaccine.

**Trial registration:**

ClinicalTrials.gov NCT04889274. Registered on 5 May 2023. The study was approved by the South Central – Oxford C Research Ethics Committee (21/SC/0154).

**Supplementary Information:**

The online version contains supplementary material available at 10.1186/s13063-023-07616-2.

## Background

Patients with pre-existing cardiovascular disease (CVD) are at greater risk of severe COVID-19 and worse outcomes [1–4]. Additionally, there has been increasing recognition of specific cardiovascular complications of COVID-19 including acute coronary syndromes and myocarditis, amongst others [5–9]. Importantly, the incidence of such cardiovascular complications is greater in men than in women [10, 11]. The mechanisms underlying these cardiovascular manifestations might be secondary to the severe inflammatory response induced by COVID-19, as well as the virus itself. These might result in endothelial dysfunction [12, 13], thus inducing hyperinflammatory and hypercoagulable states [14]. In health, the endothelium exhibits anti-inflammatory and antithrombotic properties that prevent vascular inflammation and opposes thrombogenesis [15], effects mediated predominantly by the soluble mediator nitric oxide (NO) [16–18]. In disease, the systemic inflammatory response and subsequent endothelial dysfunction reduce NO bioavailability [19]. Recently, we have demonstrated that inorganic nitrate can mitigate against typhoid vaccine-induced endothelial dysfunction, with suppression of pro-inflammatory cell types, and simultaneously enhance the resolution of inflammation with increased expression of anti-inflammatory cytokines and chemokines [20]. It is possible that the prevalence of endothelial dysfunction also contributes to the severity of COVID-19 disease.

Thus, to study the impact of systemic inflammation (related to COVID-19) upon endothelial function, in men and women, we have assessed vascular reactivity in both sexes prior to and following COVID-19 vaccination, and assessed whether augmentation of NO levels, via delivery of dietary inorganic nitrate which augments NO via activation of the non-canonical pathway [21], improves endothelial function where dysfunction is evident.

## Methods and design

### Trial objectives

Aims of research: We wish to test whether sex differences exist in inflammatory responses and thereby vascular function after delivery of the COVID-19 vaccine, as a model of endothelial dysfunction induced by COVID-19. Secondly, we also wish to determine whether inorganic nitrate supplementation protects against endothelial dysfunction in this COVID-19 setting.

### Participant selection

This is a single-centre study and participants will be recruited from the Barts Health NHS Trust catchment area, including all NHS-approved vaccination hubs. In addition, we will identify participants from Queen Mary University of London and Barts and The London Faculty of Medicine and Dentistry.

### Original hypothesis

We hypothesise that the COVID-19 vaccine will induce inflammatory responses and thus endothelial dysfunction. However, we expect women to be protected against vascular dysfunction and hypothesise that dietary inorganic nitrate will attenuate the consequent vascular dysfunction following the delivery of the COVID-19 vaccine.

### Primary endpoints

Part A

1. Comparison between the sexes of the change in flow-mediated dilatation (FMD) from the baseline FMD response after COVID-19 vaccination

Part B

1. Comparison of change in FMD from baseline after COVID-19 vaccination following inorganic nitrate versus placebo supplementation
2. Comparison of change in plasma nitrite concentration ([NO_2_^−^]) following inorganic nitrate versus placebo supplementation

### Secondary endpoints

1. Comparison of change in peripheral markers of inflammation and leucocyte count following COVID-19 vaccination between the sexes
2. Comparison of change in peripheral markers of inflammation and leucocyte count following COVID-19 vaccination and nitrate versus placebo supplementation
3. Comparison of change in PWV from baseline following COVID-19 vaccination and nitrate versus placebo supplementation
4. Comparison of change in PWA from baseline following COVID-19 vaccination and nitrate versus placebo supplementation
5. Comparison of GTN-induced vasodilation in the brachial artery from baseline following COVID-19 vaccination and nitrate versus placebo supplementation

### Inclusion criteria

1. Healthy volunteers with either a confirmed booking or a plan to book their 1st COVID-19 vaccination or due delivery of a 2nd or booster dose of any COVID-19 vaccine within the upcoming 92 days, as per government guidelines
2. Aged 18–60
3. Volunteers willing to sign the consent form

### Exclusion criteria

1. Healthy subjects unwilling to consent
2. Pregnant or any possibility that a subject may be pregnant
3. History of any serious illnesses (such as diabetes, cardiovascular disease, respiratory diseases including asthma and chronic obstructive airways disease, autoimmune conditions, and any condition which requires treatment with medication), including recent infections or trauma
4. Subjects taking systemic medication (other than the oral contraceptive pill)
5. Subjects with self-reported use of mouthwash or tongue scrapes
6. Subjects with recent (2 weeks) or current antibiotic use
7. Subjects with a history or recent treatment of (within the last 3 months) any oral condition (excluding caries), including gingivitis, periodontitis, and halitosis
8. Subjects with a history of COVID-19 vaccination within the preceding 28 days
9. Subjects with any history of a blood-borne infectious disease such as hepatitis B or C virus or HIV

### Study design and intervention

**Fig. 1:**
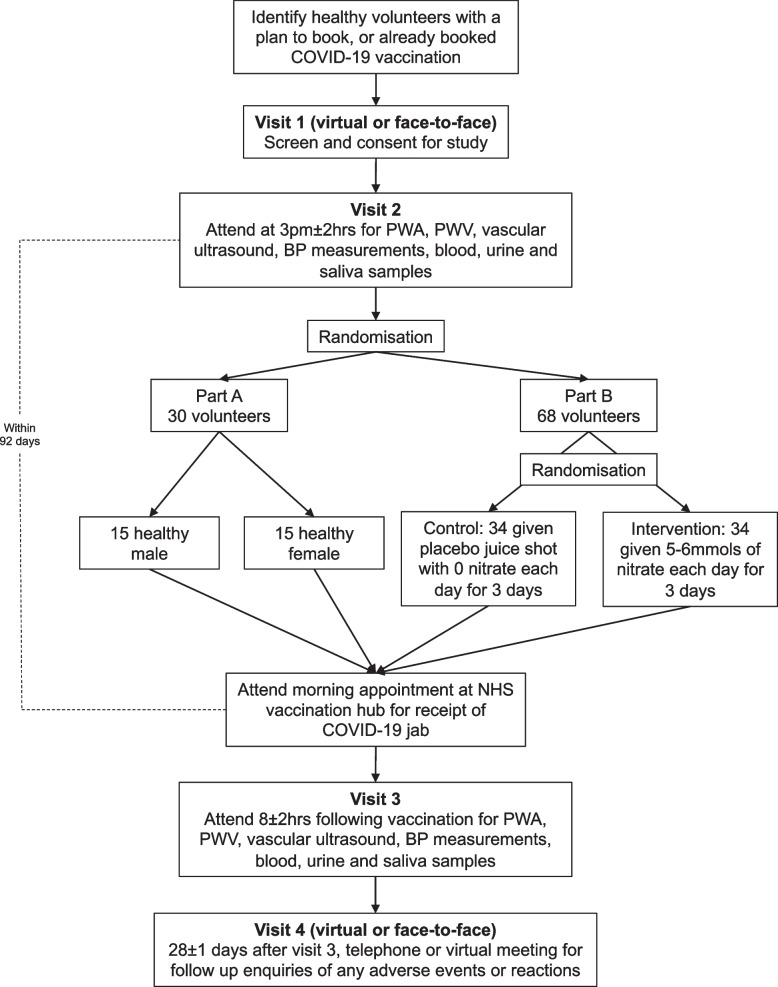
DiNOVasc-COVID-19 study summary scheme. BP, blood pressure; COVID-19, coronavirus disease 2019; NHS, National Health Service; PWA, pulse wave analysis; PWV, pulse wave velocity

**Fig. 2:**
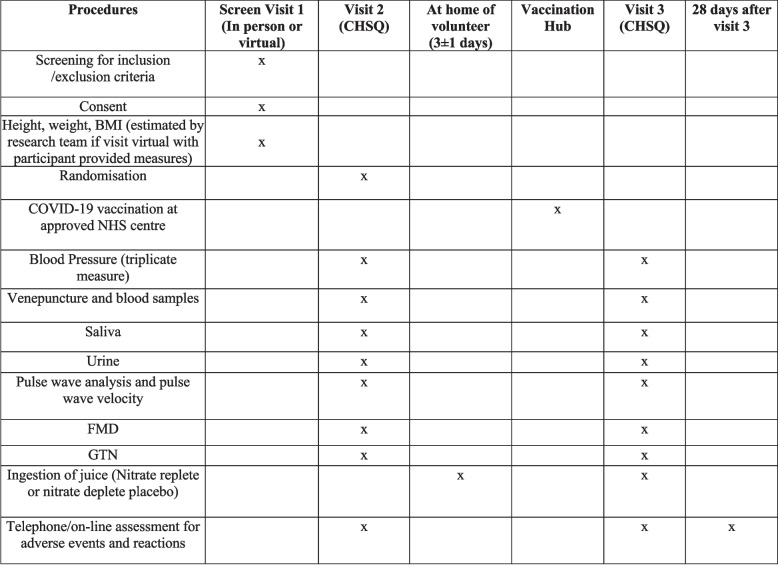
SPIRIT figure. Summary SPIRIT figure showing the visit structure and events during the clinical study

This is a prospective double-blind, placebo-controlled, clinical study. A total of 98 healthy volunteers (male and female, aged 18–60) as per inclusion criteria will be recruited following the provision of consent (model consent form added as Supplementary Document). Figure 1 shows a summary of the study scheme. Figure 2 shows the SPIRIT figure summary of the study. Participants will be screened having either booked their COVID-19 vaccination or have a plan to do so in the upcoming 92 days. Participants are able to receive any vaccine manufacturer or a combination available to them (with the manufacturer recorded in the case report form). Screening will take place virtually or in person. Participants will be randomised into 1 of 2 parts of the study. Both study parts will take place at the William Harvey Clinical Research Centre, Queen Mary University of London, Barts and The London Faculty of Medicine and Dentistry. Participants will be eligible for inclusion if they do not suffer from any illnesses that require the use of prescription medication (other than the oral contraceptive pill), and thus, the use of prescription medicines will exclude them from the study. Furthermore, following randomisation, the participant will still be eligible to undergo usual care should they require it during the study including concomitant planned and unplanned medical care (unrelated to the study itself) and medication for the time period of the physical visits and biological sampling; such an event would exclude them from the study. All events will be recorded in the case report form.

*Part A:* 15 men and 15 women will be randomised into *part A*. This part of the study will recruit equal numbers of men and women to compare the influence of biological sex on inflammatory responses and vascular function following the COVID-19 vaccine.

*Part B*: 34 men and 34 women will be randomised into *part B*. In this study, we will recruit equal numbers of men and women and make comparisons of the effects of dietary inorganic nitrate and sex on vascular function and inflammatory responses. After randomisation into *part B*, participants will undergo further randomisation to receive either 70 mL of a nitrate-containing beetroot juice (5–6 mmol, James White Drinks Ltd., UK, Beet It^®^ Sport Nitrate 400 Shot) or nitrate-deplete placebo control.

Baseline measurements of BP, PWA, PWV, FMD, blood, urine, and saliva will be taken at *visit 2*. Participants randomised to *part B* will begin taking a daily dose of the beetroot juice 2 days before receiving their COVID-19 vaccination and consume 3 consecutive daily doses. The last dose will be taken on the morning of the vaccine. All participants will receive their COVID-19 vaccination on the morning of *visit 3* and return to the clinical research facility to undergo repeat measurements of BP, PWA, PWV, FMD, blood, urine, and saliva 8 ± 2 h after the vaccine. *Visit 4* will occur 28 ± 1 days after delivery of the vaccine, at which point participants will be asked to complete a bespoke experience questionnaire. This questionnaire is bespoke and has been tailored to capture specific aspects of symptoms before and after the COVID-19 vaccination and scored from 0 to 10. These include general health, anxiety and stress, fatigue, and muscle/joint pain. We have also incorporated a rating on the experience of the intervention/placebo and included yes/no questions for whether the juice intervention/placebo was easily incorporated into their daily routine and whether the participant would be keen to continue the intervention as a lifestyle measure if found to be beneficial.

Patients will be recruited throughout all waves of the COVID-19 pandemic. Timing of when COVID-19 vaccination and previous COVID-19 infection will be obtained, to permit exploratory analyses of differences between COVID-19 waves and/or the presence of previous COVID-19 infections.

### Randomisation and blinding process

A total of 98 healthy volunteers will be recruited. *Part A* is an observational study in which 15 males and 15 females will be recruited. *Part B* is a double-blind, randomised, placebo-controlled clinical trial and will enrol 34 males and 34 females. Randomisation into *parts A* and *B* will occur in a 1:2 fashion using block randomisation, such that recruitment occurs over a similar timespan.

In *part B*, 1:1 block randomisation will be used to allocate participants to nitrate-containing beetroot juice or nitrate-deplete beetroot juice (placebo). The treatment assignment of the volunteers will remain blinded until the end of the study, at which point the study will be unblinded. If emergency unblinding is required, the chief investigator will be informed. A list of the unblinded treatment allocations will be stored in a secure location at the William Harvey Clinical Research Centre and be available at all times.

### Study start and end dates

Recruitment commenced on 21 April 2021. The provisional end date of the study is April 2024.

### Methods to be used

All sample analyses will be conducted blind to group allocation.

#### Blood, urine, and saliva analysis

Venous blood samples will be acquired, and plasma will be stored at − 80 °C for exploratory analyses. Blood samples will be sent for clinical haematological and biochemical analysis. From a separate blood sample, polymorphonuclear leucocytes (PMNs) and peripheral blood mononuclear cells (PBMCs) will be isolated.

Flow cytometry will be used to assess platelet activation and platelet leukocyte aggregates (CD42b, CD14, CD16b, CD16). Platelet P-selectin (CD62P) expression and platelet number will be determined using cell counting beads (Thermo Fisher). Platelet P-selectin expression in response to agonists, collagen (with HEPES buffer solution), and ADP will be assessed. Platelet aggregation responses to the platelet stimuli (ADP and collagen) will be assessed using a Multiplate^®^ aggregometer reader.

Total RNA will be extracted from isolated PMNs and PBMCs using a RNeasy^®^ Mini Kit according to the manufacturer’s instructions (including DNAse treatment) (Qiagen, Hilden, Germany).

Saliva samples will be centrifuged, and a pellet generated, which will contain the genetic material of the oral microbiota. A saliva sample will also be collected for human genomic sequencing using an Oragene^®^ OG-600 human saliva DNA storage kit (DNA Genotek™, Canada).

Plasma, urine, and saliva nitrate/nitrite concentration will be determined using liquid-phase ozone chemiluminescence as previously described [22, 23]. A sample of juice from each participant in *part B* will be acquired, and nitrate and nitrite quantified using the same methods. All measurements will be conducted by an individual blinded to the intervention allocations.

#### Pulse wave analysis and pulse wave velocity

PWA and PWV are non-invasive measures of arterial stiffness and compliance. PWA and PWV are quantified using a Vicorder^®^ device (Skidmore Medical Ltd., UK). PWA will be assessed by applying a cuff to the non-dominant arm, with the participant lying in the supine position. PWV will be determined by placing a cuff around both the femoral and carotid arteries simultaneously. The cuffs are inflated to ~ 65 mmHg, and the corresponding oscillometric signal from each cuff is digitally analysed to extract the pulse time delay. The aortic length is estimated by measuring the distance between the femoral cuff and the supra-sternal notch. PWV is calculated by using the formula: PWV = aortic distance/pulse time delay [24].

#### Flow-mediated dilatation

FMD is a non-invasive method of measuring endothelial function in vivo [25]. The technique uses vascular ultrasound to measure the luminal dimension of the brachial artery, which dilates following a period of limb ischaemia, owing to the release of several endothelial factors, including NO [26]. This will be performed in accordance with recently published guidelines [27]. A high-resolution external vascular ultrasound Siemens/Acuson Sequoia C256 Colour Doppler, with a 7.0-MHz linear-array transducer supported by a stereotactic clamp, will be used to image the brachial artery with the patient lying in a supine position. The brachial artery will be visualised in longitudinal section. The image is projected onto automated edge detection software (FMD Studio, Cardiovascular Suite, Quipo Srl., Italy). The edge detection algorithm tracks the vascular intima in real time providing an automated and unbiased assessment of brachial artery diameter with automated FMD calculation. A pulse-wave Doppler sample volume is placed centrally within the lumen of the vessel and calibrated to record real-time shear rate. A pneumatic cuff is placed over the upper forearm and inflated to 300 mmHg for 5 min. The cuff is then released, and the brachial artery is observed for a further 5 min. FMD is defined as the percentage increase in vessel diameter after the release of the pneumatic cuff.

Following the measurement of FMD, 0.4 mg of sublingual glyceryl trinitrate (GTN) will be administered, and the luminal diameter of the brachial artery will be measured for a period of 5 min to assess GTN-induced smooth muscle-mediated brachial artery dilatation.

#### Follow-up

At 28 ± 1 days after *visit 3*, participants will be followed up by telephone, or in person at the William Harvey Clinical Research Centre at the William Harvey Research Institute at Queen Mary University of London, to complete a brief questionnaire based upon specific aspects of symptoms.

### End of study definition

The study will end after the last participant completes *visit 4*. All samples will be analysed at the end of the study.

### Sample size determination and statistical analysis

In this study, we aim to recruit 98 participants, with 30 healthy volunteers in *part A* and 64 in *part B*. This sample size will empower the study to test the primary endpoint.

These numbers have been based upon our previous experience with typhoid vaccination which causes a reduction in FMD of ~ 1.5% absolute units which is a reduction of approximately 25% of the response [28]. We have previously identified sex differences in the response to typhoid vaccine, with a change from baseline of FMD of − 0.5 (SD = 2.4) in men and 2.4 (SD = 2.5) in women [29]. For *part A*, using these data, if we use a conservative effect size of 25% less than that achieved in our previously published study, 13 participants are required in each group to provide 90% power for the primary outcome. To account for potential dropouts of ~ 10%, 15 participants will be recruited into each group and thus 30 volunteers in total. For the analysis of *part A*, a linear regression model will be used to compare changes in vascular dysfunction pre- to post-vaccination between the sexes, unadjusted and adjusted for important risk factors including age, body mass index, and baseline vessel diameter.

Our unpublished preliminary data investigating the effects of dietary inorganic nitrate on vascular responses (FMD) has identified a decrease in FMD from baseline of 1.4 (SD = 1.5) with typhoid vaccine and with dietary nitrate intervention only 0.08 (SD = 1.7) [20]. Based on these data, for the primary outcome of change in FMD, 25 participants will be required in each group at an 80% power, to observe a difference between the intervention arms. To observe the differences between the sexes, 30 in each group provides a 90% power, which will also offer a > 95% power to detect a rise in [NO_2_^−^], as observed in previous studies [30]. To account for potential dropouts of ~ 10%, a total of 68 volunteers will be recruited. For the statistical analysis in *part B*, analysis of covariance (ANCOVA) will be used to compare the change in vascular dysfunction pre- to post-vaccination, between dietary nitrate and placebo control groups adjusting for pre-vaccination level, and to compare the change in plasma [NO_2_^−^] between the dietary nitrate and placebo control groups.

Personal data will be stored in a secured site file and clinical notes in a paper CRF, which will be security locked and coded. Only the direct research team will have access to the trial CRF. The subject will be given a unique, single identifier which will be used to identify data and samples. Results data will only be stored, shared, analysed, transmitted, and presented in an anonymised form. Participant experience questionnaires will be completed and stored in the secure CRF. Reasons for drop-out or deviation from protocol will be detailed in full. Data quality will be assessed upon completion of the study and stored electronically. Confidentiality will be maintained through these secure measures before, during, and after the trial. Data access will be granted only to direct study members. Upon completion of the study, a fully blinded data analysis will be undertaken by a statistician.

### Ethical considerations

The study protocol, participant facing documentation, recruitment and advertising material, and all amendments were submitted by the chief investigator to a Health Research Authority REC (South Central – Oxford C Research Ethics Committee, identification code 21/SC/0154). Written approval from the committee was obtained, as was sponsor approval, prior to initiating recruitment.

### Safety considerations

In *part B*, the intervention is 70 mL of concentrated beetroot juice (both nitrate-replete and placebo manufactured by James White Drinks Ltd., UK). To manufacture the placebo, an anion exchange resin is used. There are no known serious side effects related to consumption of either the intervention or placebo, and the juice is classed as a foodstuff. Recent publications support its use in clinical trials [31–33]. In the unlikely event that a serious adverse event (SAE) occurs, this will be reviewed by the chief investigator. There will be no special criteria for discontinuing or modifying the allocated intervention; however, if a decision to withdraw participants from the study is required, this will be made by the chief investigator and detailed in the final manuscript.

### Safety reporting

Any adverse event (AE) will be recorded in the participants’ case report form (CRF) and reviewed by the chief investigator. All SAEs will be reported to the sponsor (within 24 h) and REC (within 15 days), where in the opinion of the chief investigator, the event was ‘related’ (as a result of trial procedures) or ‘unexpected’ (not an expected occurrence). SAEs will be recorded in the participants’ CRF. Emergency unblinding as a result of an AE or SAE can take place at any time of day, if required.

### Patient and public involvement

The protocol underwent independent review from the local William Harvey Research Institute Peer Review Committee and review by the Queen Mary and Barts Trust Joint Research Management Office (JRMO). In addition, lay persons were invited to comment on the protocol via the NIHR Barts BRC Cardiovascular patient, public advisory group (PPAG). Furthermore, the final study documents, including participant-facing documents, underwent review and interview by the Research Ethics Council (REC) committee that includes lay members.

### Monitoring

The trial will be monitored by a Trial Steering Committee (TSC) to assess safety, feasibility, or any other arising problems. This committee will be composed of 3 independent experts in the field of pharmacology, interventional cardiology, and clinical trials, in addition to the investigators, statistician, data monitor, and a lay individual. The TSC will meet routinely during the study at 1-year intervals and will meet upon recruitment of the final participant and at study closure. The day-to-day running of the study will be conducted by the clinical fellow of the study, in conjunction with a member of the research team (clinical research assistant), both of whom report directly to the chief investigator. Representatives of Barts NIHR Cardiovascular BRC PPAG will be included in the Trial Steering Committee to be involved in oversight of the trial.

### Dissemination

Primary endpoint data analysis will begin immediately after the last study visit of the last participant. The outcomes and results of this trial will be published according to the CONSORT statement. Trial information will be communicated to the general public via the NIHR Barts Cardiovascular BRC ‘Let’s talk hearts’ seminar series. The results will be published and disseminated in peer-reviewed journals and presented at national and international conferences. DiNOVasc-COVID-19 has been registered with ClincalTrials.gov (http://clinicaltrials.gov, identification code NCT04889274).

## Discussion

In this randomised-controlled trial, we aim to assess the role of sex on vascular function and inflammatory cell trafficking in response to the COVID-19 vaccination and whether dietary inorganic nitrate alters this response. This will be the first study investigating the role of sex and inorganic nitrate treatment in COVID-19 vaccine-induced endothelial dysfunction and will offer novel mechanistic insights into the potential for dietary nitrate to be used as a therapy to attenuate endothelial injury and inflammatory cell responses after delivery of the COVID-19 vaccine and as a model of COVID-19 itself.

We envisage that the greatest barrier to the completion of this study will be recruitment in the face of a changing infection landscape and government policy regarding vaccination. Whilst there is little that the study team can do to mitigate against these issues a key aspect of the study is the randomisation of volunteers across all groups to ensure unbiased distribution of consented volunteers. Such an approach should ensure that the basic demographics and type of vaccine taken, the changing landscape of COVID-19 will be evenly distributed across the groups.

## Trial status

The current protocol version, v2.0 (21/06/2021), was amended from v1.0 with the inclusion of participants for their 2nd and booster vaccines. The first participant was recruited and included in the study in May 2021. Ninety-eight participants have undertaken their screening and second (pre-vaccine/baseline) visit. Six participants are awaiting their COVID-19 vaccine. It is expected that the last participant visit will be undertaken before the end of 2023. The SPIRIT checklist is available in Table 1.

**Table 1:** SPIRIT checklist. Summary SPIRIT table identifying key components of the study protocol

		**Reporting item**	**Page and line number**	**Reason if not applicable**
**Administrative information**				
Title	#1	Descriptive title identifying the study design, population, interventions, and, if applicable, trial acronym	Page 1, lines 4–6	
Trial registration	#2a	Trial identifier and registry name. If not yet registered, name of intended registry	Page 3, lines 1–3	
Trial registration: data set	#2b	All items from the World Health Organization Trial Registration Data Set	Page 3, line 1–3	
Protocol version	#3	Date and version identifier	Page 17, line 18	
Funding	#4	Sources and types of financial, material, and other support	Page 19, lines 2–5	
Roles and responsibilities: contributorship	#5a	Names, affiliations, and roles of protocol contributors	Page 19, line 7 to age 20 line 2	
Roles and responsibilities: sponsor contact information	#5b	Name and contact information for the trial sponsor	Page 18, lines 3–4	
Roles and responsibilities: sponsor and funder	#5c	Role of study sponsor and funders, if any, in study design; collection, management, analysis, and interpretation of data; writing of the report; and the decision to submit the report for publication, including whether they will have ultimate authority over any of these activities	Page 19, line 7 to page 20 line 2	
Roles and responsibilities: committees	#5d	Composition, roles, and responsibilities of the coordinating centre, steering committee, endpoint adjudication committee, data management team, and other individuals or groups overseeing the trial, if applicable (see Item 21a for data monitoring committee)	Page 16, lines 5–13	
**Introduction**			Page 4, lines 3–24	
Background and rationale	#6a	Description of research question and justification for undertaking the trial, including summary of relevant studies (published and unpublished) examining benefits and harms for each intervention	Page 4, lines 3–24	
Background and rationale: choice of comparators	#6b	Explanation for choice of comparators	Page 5, lines 3–7	
Objectives	#7	Specific objectives or hypotheses	Page 5, lines 9–19	
Trial design	#8	Description of trial design including type of trial (e.g. parallel-group, crossover, factorial, single group), allocation ratio, and framework (e.g. superiority, equivalence, non-inferiority, exploratory)	Page 7, line 18	
**Methods: participants, interventions, and outcomes**				
Study setting	#9	Description of study settings (e.g. community clinic, academic hospital) and list of countries where data will be collected. Reference to where list of study sites can be obtained	Page 5, lines 10–13; page 8, lines 2–3	
Eligibility criteria	#10	Inclusion and exclusion criteria for participants. If applicable, eligibility criteria for study centres and individuals who will perform the interventions (e.g. surgeons, psychotherapists)	Page 6, line 19 to page 7, line 15	
Interventions: description	#11a	Interventions for each group with sufficient detail to allow replication, including how and when they will be administered	Page 8, lines 16–19	
Interventions: modifications	#11b	Criteria for discontinuing or modifying allocated interventions for a given trial participant (e.g. drug dose change in response to harms, participant request, or improving/worsening disease)	Page 15, lines 2–10	
Interventions: adherance	#11c	Strategies to improve adherence to intervention protocols, and any procedures for monitoring adherence (e.g. drug tablet return; laboratory tests)	Page 11, line 11 to page 12, line 7	
Interventions: concomitant care	#11d	Relevant concomitant care and interventions that are permitted or prohibited during the trial	Page 8, lines 2–9	
Outcomes	#12	Primary, secondary, and other outcomes, including the specific measurement variable (e.g. systolic blood pressure), analysis metric (e.g. change from baseline, final value, time to event), method of aggregation (e.g. median, proportion), and time point for each outcome. Explanation of the clinical relevance of chosen efficacy and harm outcomes is strongly recommended	Page 10, line 12 to page 12, line 17	
Participant timeline	#13	Time schedule of enrolment, interventions (including any run-ins and washouts), assessments, and visits for participants. A schematic diagram is highly recommended (see figure)	Page 10, lines 5–6	
Sample size	#14	Estimated number of participants needed to achieve study objectives and how it was determined, including clinical and statistical assumptions supporting any sample size calculations	Page 13, line 5 to page 14, line 16	
Recruitment	#15	Strategies for achieving adequate participant enrolment to reach target sample size	Page 5, lines 10–13; page 17, lines 9–15	
**Methods: assignment of interventions (for controlled trials)**				
Allocation: sequence generation	#16a	Method of generating the allocation sequence (e.g. computer-generated random numbers), and list of any factors for stratification. To reduce predictability of a random sequence, details of any planned restriction (e.g. blocking) should be provided in a separate document that is unavailable to those who enrol participants or assign interventions	Page 9, lines 16–24; page 10, lines 1–2	
Allocation concealment mechanism	#16b	Mechanism of implementing the allocation sequence (e.g. central telephone; sequentially numbered, opaque, sealed envelopes), describing any steps to conceal the sequence until interventions are assigned	Page 9, lines 16–24; page 10, lines 1–2	
Allocation: implementation	#16c	Who will generate the allocation sequence, who will enrol participants, and who will assign participants to interventions	Page 9, lines 16–24; page 10, lines 1–2	
Blinding (masking)	#17a	Who will be blinded after assignment to interventions (e.g. trial participants, care providers, outcome assessors, data analysts), and how	Page 9, lines 16–24; page 10, lines 1–2	
Blinding (masking): emergency unblinding	#17b	If blinded, circumstances under which unblinding is permissible, and procedure for revealing a participant’s allocated intervention during the trial	Page 9, lines 16–24; page 10, lines 1–2	
**Methods: data collection, management, and analysis**				
Data collection plan	#18a	Plans for assessment and collection of outcome, baseline, and other trial data, including any related processes to promote data quality (e.g. duplicate measurements, training of assessors) and a description of study instruments (e.g. questionnaires, laboratory tests) along with their reliability and validity, if known. Reference to where data collection forms can be found, if not in the protocol	Page 14, lines 7–16	
Data collection plan: retention	#18b	Plans to promote participant retention and complete follow-up, including list of any outcome data to be collected for participants who discontinue or deviate from intervention protocols	Page 14, lines 7–16	
Data management	#19	Plans for data entry, coding, security, and storage, including any related processes to promote data quality (e.g. double data entry; range checks for data values). Reference to where details of data management procedures can be found, if not in the protocol	Page 14, lines 7–16	
Statistics: outcomes	#20a	Statistical methods for analysing primary and secondary outcomes. Reference to where other details of the statistical analysis plan can be found, if not in the protocol	Page 13, line 6 to page 14, line 16	
Statistics: additional analyses	#20b	Methods for any additional analyses (e.g. subgroup and adjusted analyses)	Page 13, line 6 to page 14, line 16	
Statistics: analysis population and missing data	#20c	Definition of analysis population relating to protocol non-adherence (e.g. as randomised analysis), and any statistical methods to handle missing data (e.g. multiple imputation)	Page 13, line 6 to page 14, line 16	
**Methods: monitoring**				
Data monitoring: formal committee	#21a	Composition of data monitoring committee (DMC); summary of its role and reporting structure; statement of whether it is independent from the sponsor and competing interests; and reference to where further details about its charter can be found, if not in the protocol. Alternatively, an explanation of why a DMC is not needed	Page 15, lines 13–18; page 16, lines 5–13	
Data monitoring: interim analysis	#21b	Description of any interim analyses and stopping guidelines, including who will have access to these interim results and make the final decision to terminate the trial	Page 15, lines 13–18; page 16, lines 5–13	
Harms	#22	Plans for collecting, assessing, reporting, and managing solicited and spontaneously reported adverse events and other unintended effects of trial interventions or trial conduct	Page 15, lines 2–18	
Auditing	#23	Frequency and procedures for auditing trial conduct, if any, and whether the process will be independent from investigators and the sponsor	Page 15, lines 13–18; page 16, lines 5–13	
**Ethics and dissemination**				
Research ethics approval	#24	Plans for seeking research ethics committee/institutional review board (REC/IRB) approval	Page 14, lines 19–23	
Protocol amendments	#25	Plans for communicating important protocol modifications (e.g. changes to eligibility criteria, outcomes, analyses) to relevant parties (e.g. investigators, REC/IRBs, trial participants, trial registries, journals, regulators)	Page 14 lines 19–23; page 17, line 18; page 18, lines 3–8	
Consent or assent	#26a	Who will obtain informed consent or assent from potential trial participants or authorised surrogates, and how (see item 32)	Page 18, lines 8–12	
Consent or assent: ancillary studies	#26b	Additional consent provisions for collection and use of participant data and biological specimens in ancillary studies, if applicable	Page 7, line 20	
Confidentiality	#27	How personal information about potential and enrolled participants will be collected, shared, and maintained in order to protect confidentiality before, during, and after the trial	Page 14, lines 7–16	
Declaration of interests	#28	Financial and other competing interests for principal investigators for the overall trial and each study site	Page 18, line 22	
Data access	#29	Statement of who will have access to the final trial dataset, and disclosure of contractual agreements that limit such access for investigators	Page 16, lines 5–13	
Ancillary and post-trial care	#30	Provisions, if any, for ancillary and post-trial care, and for compensation to those who suffer harm from trial participation	Page 15, lines 2–18	
Dissemination policy: trial results	#31a	Plans for investigators and sponsor to communicate trial results to participants, healthcare professionals, the public, and other relevant groups (e.g. via publication, reporting in results databases, or other data sharing arrangements), including any publication restrictions	Page 16, lines 16–22	
Dissemination policy: authorship	#31b	Authorship eligibility guidelines and any intended use of professional writers	Page 16, lines 16–22	
Dissemination policy: reproducible research	#31c	Plans, if any, for granting public access to the full protocol, participant-level dataset, and statistical code	Page 16, lines 16–22	
**Appendices**				
Informed consent materials	#32	Model consent form and other related documentation given to participants and authorised surrogates	Page 7, line 20. Supplementary Material	
Biological specimens	#33	Plans for collection, laboratory evaluation, and storage of biological specimens for genetic or molecular analysis in the current trial and for future use in ancillary studies, if applicable	Page 18, lines 11–12	

### Supplementary Information

**Additional file 1.** Participant consent form.

## Data Availability

The supplementary material of the final manuscript will contain the full protocol, deidentified dataset, and statistical code.
