# Supplementary material for: A double-blind, randomised, placebo-controlled parallel study to investigate the effect of sex and dietary nitrate on COVID-19 vaccine-induced vascular dysfunction in healthy men and women: protocol of the DiNOVasc-COVID-19 study
Source: Trials. 2023 Sep 16;24:593. doi: 10.1186/s13063-023-07616-2 (PMC10504715; doi:10.1186/s13063-023-07616-2)
Supplement: Supplementary file 1 — Additional file 1. Participant consent form. [file 13063_2023_7616_MOESM1_ESM.pdf]

Queen Mary University of London

NHS Barts Health NHS Trust

CONSENT FORM

Participant Identification Number for this trial:……………………………

Title of Project: A double-blind, randomised, placebo-controlled parallel study to investigate the effect of dietary nitrate and sex on COVID-19 vaccine induced vascular dysfunction in healthy men and women.

Short title: DiNOVasc-COVID-19

Please initial

1. I confirm that I have read the information sheet dated................... (version............) for the above study. I have had the opportunity to consider the information, ask questions and have had these answered satisfactorily. ☐
2. I understand that my participation is voluntary and that I am free to withdraw at any time without giving any reason, without my medical care or legal rights being affected. ☐
3. I understand that relevant sections of my medical notes and data collected during the study, may be looked at by individuals from regulatory authorities or from the NHS Trust, where it is relevant to my taking part in this research. I give permission for these individuals (including the sponsor) to have access to my records. ☐
4. I understand that the information collected about me will be used to support other research in the future, and I consent for blood, urine, and saliva to be collected and processed for analysis including DNA isolation for genetic analyses as part of this research study. I understand samples are to be stored for 3 years following end of the study and my information for up to 25 years and may be shared anonymously with other researchers. ☐
5. I agree to my General Practitioner being informed of my participation in the study. I agree to my General Practitioner being involved in the study, including any necessary exchange of information about me between my GP and the research team. ☐
6. I agree to take part in the above study. ☐

______________________	______________	______________
Name of Participant	Date	Signature
______________________	______________	______________
Name of person taking consent	Date	Signature

7. I consent to future contact by the research team for potential future study participation. ☐

______________________	______________	______________
Name of Participant	Date	Signature
______________________	______________	______________
Name of person taking consent	Date	Signature
